# Geographic distribution and genetic diversity of the *Ehrlichia *sp. from Panola Mountain in *Amblyomma americanum*

**DOI:** 10.1186/1471-2334-8-54

**Published:** 2008-04-23

**Authors:** Amanda D Loftis, Tonya R Mixson, Ellen Y Stromdahl, Michael J Yabsley, Laurel E Garrison, Phillip C Williamson, Robert R Fitak, Paul A Fuerst, Daryl J Kelly, Keith W Blount

**Affiliations:** 1 Centers for Disease Control and Prevention, Atlanta, GA 30333, USA; 2 U.S. Army Center for Health Promotion and Preventive Medicine, Entomological Sciences Program, Aberdeen Proving Ground, MD 21010-5403, USA; 3 Daniel B. Warnell School of Forestry and Natural Resources, University of Georgia, Athens, GA 30602, USA; 4 Southeastern Cooperative Wildlife Disease Study, Department of Population Health, College of Veterinary Medicine, University of Georgia, Athens, GA 30602, USA; 5 Georgia Division of Public Health, Atlanta, GA 30303-3186, USA; 6 DNA/Identity Laboratory, Department of Pathology and Human Identification, University of North Texas Health Science Center, Ft. Worth, TX 76107-2699, USA; 7 Department of Evolution, Ecology and Organismal Biology, The Ohio State University, Columbus, OH 43210-1293, USA; 8 Air Force Research Laboratory, Brooks City-Base, TX 78235, USA; 9 266 N. Lincoln St., Laramie, WY 82070, USA

## Abstract

**Background:**

A novel *Ehrlichia*, closely related to *Ehrlichia ruminantium*, was recently discovered from Panola Mountain State Park, GA, USA. We conducted a study to determine if this agent was recently introduced into the United States.

**Methods:**

We developed a sensitive PCR assay based on the conserved *glt*A (citrate synthase) gene and tested DNA samples extracted from 1964 field-collected and 1835 human-biting *Amblyomma americanum *from 23 eastern states of the USA.

**Results:**

The novel agent was detected in 36 ticks collected from 10 states between 1998 and 2006. Infected ticks were collected both from vegetation (n = 14, 0.7%) and from humans (n = 22, 1.2%). Fragments of the conserved *glt*A gene and the variable *map*1 gene were sequenced from positive samples. Two distinct clades, with 10.5% nucleic acid divergence over the 730 bp *map*1 sequence, were identified.

**Conclusion:**

These data suggest that the Panola Mountain *Ehrlichia *was not recently introduced to the United States; this agent has an extensive distribution throughout the range of its tick vector, has been present in some locations for several years, and displays genetic variability. Furthermore, people in several states were exposed to this agent through the bite of infected ticks, underscoring the potential public health risk of this emerging ehrlichiosis.

## Background

A novel *Ehrlichia *transmitted by *Amblyomma americanum *(lone star ticks) was recently discovered in Panola Mountain State Park, Georgia, USA. The "Panola Mountain *Ehrlichia*" (PME), which is closely related to *E. ruminantium*, caused transient febrile illness, followed by chronic latent infection, in a goat [1]. This agent was also associated, using PCR and sequencing, with a case of human illness following a bite from a nymphal *Amblyomma *acquired at Panola Mountain State Park [2]. White-tailed deer (*Odocoileus virginanus*) are a probable vertebrate reservoir for PME in the United States; deer are susceptible to infection, are naturally exposed to the agent, and are competent reservoirs for tick transmission of this agent [3].

*Ehrlichia ruminantium *is endemic in southern Africa and the Caribbean, and its pathogenicity in cattle, sheep, and goats varies from mild febrile illness to fatal heartwater [4-7]. Several species of *Amblyomma *vector *E. ruminantium *in Africa and *A. variegatum *is the vector in the Caribbean [4]. This exotic disease has not been detected in the United States, but it could be introduced into the country by the importation of animals and tick vectors from endemic areas [8,9]. White-tailed deer are also susceptible to infection with *E. ruminantium *[10], and could provide a sylvatic reservoir for *E. ruminantium *in the United States. Thus, the *Ehrlichia *discovered at Panola Mountain State Park could be a divergent strain of *E. ruminantium *that was recently introduced to this country. This park is located within the Atlanta, Georgia, metropolitan area and is less than twenty miles from the Hartsfield International Airport, a port of entry for imported animals and animal products.

To evaluate the geographic distribution and public health risk posed by this emerging agent, we developed a sensitive nested PCR assay based on the *glt*A (citrate synthase) gene, which is conserved within species of *Ehrlichia *[11], and screened DNA extracts from *A. americanum *collected from vegetation and submitted through four different human-biting tick surveillance programs. All PCR amplicons were confirmed by sequencing the *glt*A fragment, and the genetic diversity of this novel agent was evaluated by amplification and sequencing of the variable Major Antigenic Protein 1 gene (*map*1) [12].

## Methods

### Collection of ticks

Ticks were collected in the years 1998–2006. Questing ticks were collected by flagging vegetation or by using carbon dioxide baited traps, as described by Fleetwood et al. [13]. Ticks removed from humans were collected through surveillance programs established by the Georgia Division of Public Health, Ohio Department of Health, University of North Texas Health Science Center, and U.S. Army Center for Health Promotion and Preventive Medicine. Ticks were voluntarily submitted by individuals who reported the location in which they believed the tick was acquired. All ticks were identified to species and life stage, and gender of adult ticks was recorded. DNA was extracted from individual questing adult ticks as previously described [1,14]. For human-biting tick collections, DNA was extracted from individual nymphal and adult ticks, or from pools of ticks collected from a single person, using previously described methods [15].

### PCR detection of PME

A nested PCR assay for the Panola Mountain *Ehrlichia *was designed based on the published *glt*A sequence (GenBank:DQ363995). Nested PCR primers were designed using Primer Express 2.0 (Applied Biosystems, Foster City, CA) and GCG Seqlab (Accelrys, San Diego, CA), and evaluated for specificity using a panel of DNA from *A. americanum *known to be PCR-positive for the agent and negative DNA samples extracted from colony-reared ticks. Sensitivity was determined using a cloned plasmid containing the *glt*A gene from the *Ehrlichia *sp. Reaction conditions were optimized to ensure a sensitivity of at least 10 gene copies. The outer PCR reaction included 5.0 μL of Taq Master Mix (Qiagen, Valencia, CA), 500 nM each of primers Ehr3CS-185F (5'-GCC ACC GCA GAT AGT TAG GGA) and Ehr3CS-777R (5'-TTC GTG CTC GTG GAT CAT AGT TTT), and 1.0–2.0 μL of DNA in a 10 μL final reaction volume. The volume of DNA used in each reaction was based upon the elution volume of DNA; 1.0 μL was used for DNA samples in a final volume of 50–100 μL, and 2.0 μL were used for samples extracted in a volume of 200 μL. The thermocycler program was as follows: 95°C for 3 min, followed by 40 cycles of 95°C for 30 sec, 55°C for 30 sec, and 72°C for 60 sec, with a final extension at 72°C for 5 min. The inner PCR reaction included 10.0 μL of Taq Master Mix, 500 nM each of primers Ehr3CS-214F (5'-TGT CAT TTC CAC AGC ATT CTC ATC) and Ehr3CS-619R (5'-TGA GCT GGT CCC CAC AAA GTT), and 1.5 μL of the primary reaction product in a 20 μL final reaction volume. The thermocycler program was similar to the primary reaction, but with a 60°C annealing temperature rather than 55°C.

All PCR reactions were prepared in a dedicated hood equipped with an ultraviolet light source. Positive (10 copy) and negative controls were included on every plate; positive control material was handled only after all unknown samples were loaded. Ticks that were identified as positive using the *glt*A primer set were confirmed by repeating the assay and by sequencing. Positive ticks were tested using a PCR assay for *map*1, as previously described [1,16], using the outer primer pair map1-forward/map1-reverse and, if needed, paired heminested reactions with Pmap-2F/map1-reverse and map1-forward/Pmap-2R or nested reactions with Pmap-2F/Pmap-2R.

### Sequence analysis

All PCR amplicons obtained using the *glt*A assay and *map*1 assay were sequenced, with 2- to 4- fold coverage, using PCR primers. When mixed sequences were obtained, PCR amplicons were cloned using TOPO TA Cloning kits with the pCR 2.1-TOPO vector (Invitrogen, Carlsbad, CA) and sequencing was repeated using several clones. Fragments were assembled using GCG Seqmerge (Accelrys), translated into the respective predicted amino acid sequences, and aligned. All sequences were submitted to GenBank: *glt*A, EU272374–EU272410 and *map*1, EU272339–EU272373. Related sequences were identified using BLAST (NCBI, Bethesda, MD). Phylogenetic reconstructions based on the amino acid sequences of the *map*1 amplicons were performed using PAUP 4.0 Beta 10 (Sinauer Asosciates, Inc., Sunderland, MA). The most parsimonious trees were constructed using heuristic bootstrap analysis (1000 replicates); starting trees were obtained by stepwise addition with tree-bisection-reconnection as the branch-swapping algorithm.

## Results and Discussion

**Figure 1:**
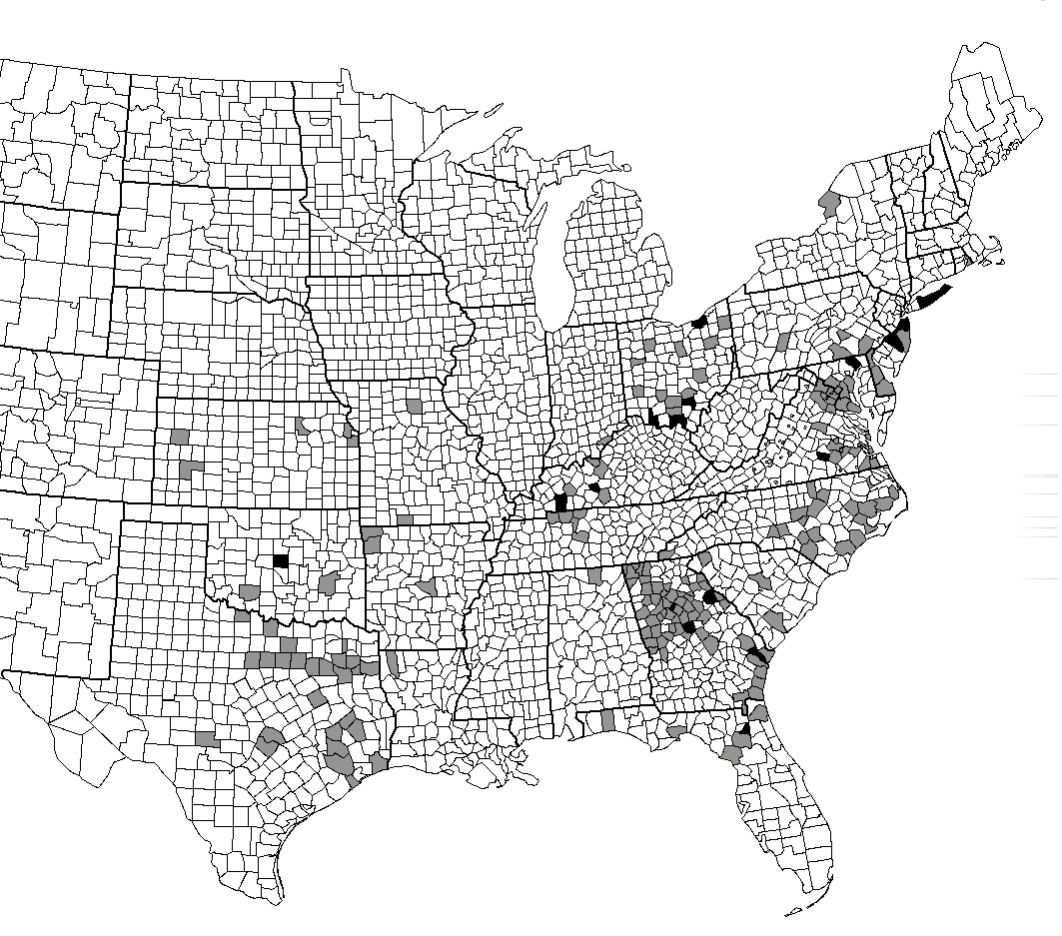
**Geographic distribution of the Panola Mountain Ehrlichia sp**. Summary of collection sites for *Amblyomma americanum*, by county. Counties from which ticks were tested and PME was not detected are shaded gray (n = 185); counties from which at least one tick contained DNA from PME are shaded black (n = 18). In addition to the counties listed in Table 3, Rockdale County, Georgia, which contains Panola Mountain State Park, is shaded black.

*Amblyomma americanum *were collected from 203 counties in 23 states, representing much of the geographic range of this tick (Figure 1).

Questing ticks were collected from vegetation at 32 sites in 8 states. Of 1964 questing adult ticks, including 867 adult males and 1097 adult females, 14 ticks (10 males, 1.15%, and 4 females, 0.36%) were positive for PME (Table 1). The overall prevalence of infection in questing ticks was 0.71%, and the prevalence of infection in sites with infected ticks ranged from 1.32–2.88%.

**Table 1:** Summary of test results for ticks collected from vegetation. *Amblyomma americanum *adults were collected from vegetation and tested for the presence of the Panola Mountain *Ehrlichia *sp.

**Collection State**	**# Ticks (M/F)**	**# POS (M/F)**	**% POS**
Florida	151 (57/94)	1 (1/0)	0.66%
Georgia	705 (292/413)	6 (3/3)	0.85%
Kentucky	6 (1/5)	1 (0/1)	16.7%
New Jersey	120 (68/52)	2 (2/0)	1.67%
New York	475 (246/229)	4 (4/0)	0.84%
North Carolina	383 (152/231)	0	0%
South Carolina	80 (37/43)	0	0%
Texas	44 (14/30)	0	0%

TOTAL	1964 (867/1097)	14 (10/4)	0.71%

Results are summarized here by state of collection and gender (M = male, F = female).

Human-biting ticks were obtained, via surveillance programs, from 21 states. Of 1835 *A. americanum *obtained from surveys of human-biting ticks, 22 DNA samples from 5 states were positive (Table 2). Six of these positive samples were pools of 2–9 ticks, with the remainder representing individual ticks. Given the low prevalence of infection, we assumed that only one tick in a positive pool was likely to be positive; the infection rate among human-biting ticks was therefore 22/1835, or 1.20%. Eleven of the positive samples were from nymphal ticks (1.12%), three from males (0.71%), five from females (1.17%), and five from pools containing more than one life stage.

**Table 2:** Summary of test results for human-biting ticks. *Amblyomma americanum *nymphs and adults were collected from human-biting tick surveillance programs and tested for the presence of the Panola Mountain *Ehrlichia *sp. Results are summarized here by the state of reported tick acquisition and life stage or gender (N = nymph, M = male, F = female).

**Collection State**	**# Ticks (N/M/F/Not Recorded)**	**# POS (N/M/F/unknown)^a^**	**% POS**
Alabama	8 (2/2/4/0)	0	
Arkansas	8 (1/2/4/1)	0	
District of Columbia	7 (1/3/3/0)	0	
Delaware	12 (8/2/2/0)	0	
Florida	7 (3/2/1/1)	0	
Georgia	343 (157/87/98/1)	0	
Indiana	1 (1/0/0/0)	0	
Kansas	67 (32/12/22/1)	0	
Kentucky/Tennessee	199 (114/40/45/0)	1 (0/0/1/0)	0.50%
Louisiana	1 (1/0/0/0)	0	
Maryland	266 (178/51/37/2)	4 (3/1/0/0)	1.50%
Missouri	10 (3/3/4/0)	1 (0/0/1/0)	10.0%
North Carolina	93 (45/25/23/0)	0	
Nebraska	1 (1/0/0/0)	0	
New Jersey	265 (163/57/45/3)	7 (3/1/1/2^a^)	2.64%
New York	1 (1/0/0/0)	0	
Ohio	22 (11/7/4/0)	6 (3/1/2/0)	27.3%
Oklahoma	21 (14/3/3/1)	1 (0/0/0/1^a^)	4.76%
Pennsylvania	4 (1/1/2/0)	0	
Rhode Island	1 (0/0/1/0)	0	
South Carolina	33 (17/7/9/0)	0	
Texas	81 (23/32/26/0)	0	
Virginia	368 (199/80/89/0)	1 (1/0/0/0)	0.27%
Unknown/Unrecorded	16 (7/5/4/0)	1 (1/0/0/0)	6.25%

TOTAL	1835 (983/421/426/10)	22 (11/3/5/3)	1.20%

a: When a pool of ticks (2–9 ticks each) was positive, the minimum infection rate (1 tick) is reported. The life stage of the infected tick could not be determined for 3 pools that included more than one life stage.

b: Kentucky and Tennessee ticks were combined, since most of the ticks in this collection centered on the border between these two states.

Overall, 35 positive ticks were obtained from 18 counties in ten states: Florida, Georgia, Kentucky, Maryland, Missouri, New Jersey, New York, Ohio, Oklahoma, and Virginia (Figure 1). Four of these ticks were co-infected with *E. chaffeensis *and/or *E. ewingii *(Table 3). None of the positive ticks were co-infected with "*Borrelia lonestari*". All of the positive ticks were collected between the months of April and September. Positive ticks were identified from collections made in 1998 through 2006; on Fire Island, in Suffolk Co., New York, individual positive ticks were collected from vegetation in both 1998 and 2003, suggesting that the agent was maintained in this area over several years. Similarly, positive human-biting ticks in Burlington Co., New Jersey were collected in 2001 and 2006, and ticks collected from Scioto Co., Ohio in 2000 and 2001 were positive.

**Table 3:** Collection details and map1 sequence homologies of the positive ticks. Collection details for the 36 *Amblyomma americanum *harboring DNA from the Panola Mountain *Ehrlichia *sp. (PME) are shown, including the source of the tick, date of collection, coinfectionn status, and homology of the *map*1 DNA amplicons to reference sequences for PME.

**Location Tick Was Collected or Acquired**		**Collected From**	**Life Stage**	**Date Collected**	**Coinfection with Other *Ehrlichia***	***map*1 GenBank Sequence ID**	**map1 Homology^a^**
FL	Bradford Co.	vegetation	M	6/25/2003		EU272339	730/730
GA	Jones Co.	vegetation	F	6/9/2003		EU272340	730/730
	Jones Co.	vegetation	M	6/9/2003		EU272341	654/730
	Jones Co.	vegetation	M	6/9/2003		EU272342	654/730
	Wilkes Co.	vegetation	M	6/11/2004		EU272343	730/730
	Wilkes Co.	vegetation	F	6/11/2004		EU272344	730/730
	Bryan Co.	vegetation	F	6/18/2004		EU272345	730/730
KY	Edmonson Co.	vegetation	F	6/30/2002		EU272346	654/730
	Christian Co.	22 yo female	2 FF	6/13/2006		EU272347	654/730
MD	Harford Co.	20 yo male	N	7/18/2001		EU272348	384/384^b^
	Harford Co.	52 yo male	N	5/31/2006		EU272349	730/730
	Harford Co.	25 yo male	M	6/7/2006		EU272350	730/730
	Harford Co.	50 yo male	9 NN	6/28/2006	*E. ewingii*	no amplification	
MO	Not recorded	human^c^	F	6/23/2000		no amplification	
NJ	Burlington Co.	35 yo male	N	6/29/2001		EU272351	384/384^b^
	Burlington Co.	34 yo male	F	6/29/2001		EU272352	277/277^b^
	Monmouth Co.	vegetation	F	4/22/2003	*E. ewingii E. chaffeensis*	EU272353	654/730
	Monmouth Co.	vegetation	F	4/22/2003		EU272354	730/730
	Ocean Co.	41 yo male	M+N	5/22/2006	*E. chaffeensis*	EU272355	654/730
	Ocean Co.	46 yo male	F+N	6/27/2006		EU272356–EU272360^d^	730/730, 654/730^d^
	Burlington Co.	41 yo male	M	7/20/2006		EU272361	730/730
	Burlington Co.	31 yo male	N	7/20/2006		EU272362	730/730
	Burlington Co.	46 yo male	2 NN	8/1/2006		EU272363	730/730
NY	Suffolk Co.	vegetation	M	7/3/1998	*E. ewingii E. chaffeensis*	EU272364	730/730
	Suffolk Co.	vegetation	M	6/4/2003		EU272365	730/730
	Suffolk Co.	vegetation	M	6/4/2003		EU272366	730/730
	Suffolk Co.	vegetation	M	7/9/2003		EU272367	730/730
OH	Scioto Co.	human^c^	F	5/8/2000		EU272368	377/377^b^
	Clermont Co.	human^c^	M	5/30/2000		EU272369	384/384^b^
	Cuyahoga Co.	human^c^	F	6/7/2000		EU272370	375/375^b^
	Vinton Co.	human^c^	N	6/23/2000		no amplification	
	Hocking Co.	human^c^	N	9/13/2000		EU272371	376/377^b^
	Scioto Co.	human^c^	N	6/27/2001		EU272372	313/313^b^
OK	Oklahoma Co.	human^c^	F+7NN	7/25/2006		EU272373	730/730
VA	Nottoway Co.	25 yo male	N	6/27/2006		no amplification	
State not recorded		human^c^	N	5/23/2000		no amplification	

a: Percent homology to the reference sequence for PME, GenBank: DQ324368

b: Only the internal, nested fragment was available for sequencing.

c: Age and gender not recorded.

d: The *map*1 PCR amplicon was a mixture of more than one sequence, and the product was cloned prior to sequencing.

The nested *glt*A amplicon was sequenced from all positive ticks. All positive amplicons had sequences consistent with PME; *E. chaffeensis *or *E. ewingii *were not amplified. The *glt*A sequence was highly conserved and was identical to the reported PME sequence (GenBank: DQ363995) in 35/36 ticks; one sequence included a single nucleotide polymorphism (EU272407).

A portion of *map*1 was successfully amplified and sequenced from 31 of the 36 positive tick DNA samples; the outer PCR amplicon, when available, produced 730 bp of sequence, and the inner amplicon produced 384 bp. Two *map*1 genogroups were identified and were seen both in questing ticks and in human-biting ticks (Table 3): one group was 100% homologous to the sequence reported from PME (GenBank:DQ324368), and the other group was 89.5% similar to PME, with two polymorphic loci identified within this group of sequences. There were five base pair mismatches between the sequences in the latter genogroup and the outer pair of PCR primers previously developed for the detection of the *map*1 gene from this agent (Pmap-38F/Pmap-581R, [1]). The two different PME clades were not geographically isolated; both clades were detected in ticks from Jones County, Georgia and Monmouth County, New Jersey. Additionally, one pool of ticks from Ocean County, New Jersey, contained both genotypes. The amplicons produced from this mixed template pool were cloned and sequenced. Sequence analysis indicated representatives of both genotypes were present, as well as several sequences that appear to be combinations of these two genotypes and may indicate a crossover event in *map*1 (GenBank: EU272356–EU272360).

**Figure 2:**
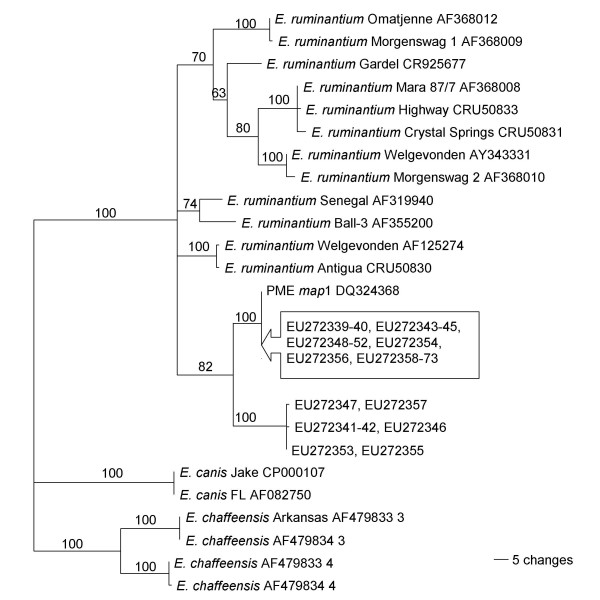
**Genetic diversity of the Panola Mountain Ehrlichia sp**. Phylogenetic reconstruction of the *map*1 predicted amino acid sequences from 31 *Amblyomma americanum *harboring DNA from the Panola Mountain *Ehrlichia *sp. (PME). Numbers indicate the bootstrap support for each node, as a percentage of 1000 replicates, and the scale represents the number of changes per 100 residues.

Phylogenetic reconstructions based on the *map*1 amino acid sequences were attempted (Figure 2) using all of the DNA sequences that were 730 bp in length. The sequences from the ticks formed two distinct clades, with 100% bootstrap support for each clade (1000 psuedoreplicates), and they formed a cluster within the larger taxonomic group of *E. ruminantium *strains in 82% of the replicates. The PME/*E. ruminantium *clade was a sister taxa to *E. chaffeensis *and *E. canis *in 100% of the replicates. Bootstrap support for separation of PME and *E. ruminantium *into separate taxa was weak (< 500/1000).

## Conclusion

We detected the novel Panola Mountain *Ehrlichia *sp. (PME), recently discovered in *A. americanum *from north central Georgia, USA, in 36 ticks from 18 counties in ten states. DNA from PME was detected in ticks from the traditional southeastern range of *A. americanum *as well as from recently established populations in the northeastern USA. The overall prevalence of infection with PME, approximately 1–3%, was similar to that previously reported from Panola Mountain [1]. Similar prevalences of *E. ewingii *and *E. ruminantium *are seen in populations of *Amblyomma *infected with these agents [14,17-19].

Earlier assays for PME were based on the gene encoding the Major Antigenic Protein 1 (*map*1) [1]; this gene can be highly variable [12], and polymorphisms in the primer annealing sites could prevent the detection of some genotypes of PME. We therefore developed a sensitive and specific nested PCR assay based on the conserved *glt*A gene. As expected, *glt*A amplicons obtained from PME-infected ticks were 99.5–100% identical to each other and to the sequence previously reported from this agent. Significant mismatches were seen between the previously reported *map*1 primers and the *map*1 sequences from PME-infected ticks. Additionally, *map*1 could not be amplified from all of the *glt*A-positive ticks, presumably due to primer site mismatches, confirming that detection of PME using *map*1 assays is limited by the variability of this gene.

This gene is, however, a valuable tool to assess genetic variability in PME across the geographic range of *A. americanum*. Using phylogenetic reconstruction of *map*1 sequences, we identified two clades, one of which includes the genotype previously reported from Panola Mountain. Both PME clades were closely associated with each other and were more closely related to *E. ruminantium *than to any other species of *Ehrlichia*, similar to previous reports. Sequences that suggest the possibility of crossover between these two genotypes were also obtained from a few clones from a single DNA sample; collection and analysis of other specimens, to determine if this is an isolated finding, are ongoing.

The extensive geographic distribution of PME and the presence of genetic variability within the species suggest that this agent was not recently introduced to the United States. A recent point source introduction should have resulted in a limited geographic distribution of a single genotype or possibly of closely related genotypes with minimal divergence. Although two distinct genotypes of PME were detected, less genetic diversity was seen than is reported to occur between African strains of *E. ruminantium*, suggesting that the *map*1 gene of PME might evolve more slowly than that of its African counterparts, that the diversity of susceptible *Amblyomma *and vertebrate species in Africa might have contributed to the diversity of *E. ruminantium *on that continent, or that the introduction of *E. ruminantium*-like bacteria to North America might have been more recent than the introduction into Africa.

Finally, the lack of reports of heartwater, or heartwater-like disease, in ruminants, wild or domestic, from areas in which PME was detected provides indirect support that this agent has low pathogenicity for these animals. However, this emerging ehrlichiosis has been associated with a human case of illness following the bite of a nymphal *A. americanum *from Panola Mountain State Park. *Amblyomma americanum *is an abundant and aggressive human-biting tick throughout its geographic range [20]. Our detection of PME in 22 human-biting ticks underscores the potential public health risk of this emerging tick-transmitted disease. The broad geographic range and patchy distribution of ticks harboring PME suggests that human cases could occur throughout the eastern United States but be sporadic in nature and therefore difficult to diagnose. Sensitive and specific PCR assays, performed on whole blood samples collected during the acute febrile period, might assist in the rapid clinical diagnosis of undifferentiated ehrlichial infections in people with a history of tick bite by *A. americanum*.

## Competing interests

The authors declare that they have no competing interests.

## Authors' contributions

AL conceived of the study, carried out portions of the molecular genetic studies, participated in the sequence alignment and analysis, performed statistical analysis, and drafted the manuscript. TM, ES, and KB participated in the design of the study, collected tick samples, carried out portions of the molecular genetic studies, and helped to draft the manuscript. PW participated in the design of the study, collected tick samples, carried out portions of the molecular genetic studies, participated in the sequence analysis, and helped to draft the manuscript. MY and RF carried out portions of the molecular genetic studies, participated in the sequence alignment, and helped to draft the manuscript. LG, PF, and DK participated in the design of the study, collected tick samples, and helped to draft the manuscript. All authors read and approved the final manuscript.

## Pre-publication history

The pre-publication history for this paper can be accessed here:


